# Growth Failure and Delayed Puberty in an Adolescent with Previously Undiagnosed Advanced HIV Infection: A Case Report

**DOI:** 10.3390/reports9040352

**Published:** 2026-10-14

**Authors:** Nikolay Balgaranov, Teodora Marinova-Bulgaranova, Stanimira Elkina

**Affiliations:** 1 Department of Pediatrics, Medical University—Pleven, 5800 Pleven, Bulgaria; 2 Department of Microbiology and Virology, Medical University—Pleven, 5800 Pleven, Bulgaria

**Keywords:** HIV infection, growth failure, delayed puberty, SARS-CoV-2 infection, late diagnosis, adolescent

## Abstract

**Background and Clinical Significance:** Longstanding human immunodeficiency virus (HIV) may remain clinically unrecognized until adolescence. Growth failure, delayed puberty, and recurrent or atypical infections may represent manifestations of underlying immunodeficiency but can initially be attributed to other conditions. **Case Presentation:** A 16-year-old male, adopted at 4 years of age, was admitted with prolonged low-grade fever, chronic productive cough, and recurrent sinonasal symptoms. Marked growth retardation and absent pubertal development were also present. No perinatal or early-childhood medical records were available, and maternal HIV status could not be established. Laboratory investigations demonstrated severe CD4+ T-cell depletion, hypergammaglobulinemia, and elevated liver enzymes. Repeated SARS-CoV-2 antigen/PCR tests were positive, while anti-SARS-CoV-2 antibodies were undetectable. CT demonstrated chronic pansinusitis and hepatosplenomegaly. HIV infection was confirmed by positive ELISA and Western blot, with a plasma HIV RNA level of 127,721 copies/mL. The findings were consistent with advanced HIV infection with severe immunosuppression. Although the clinical history suggested longstanding infection, the timing and route of HIV acquisition could not be established. Similarly, the available SARS-CoV-2 results did not allow definitive differentiation between persistent infection and separate infection or reinfection episodes. Previous CMV IgG positivity had contributed to an earlier attribution of the hepatic abnormalities to CMV, although active CMV disease was not documented. **Conclusions:** Severe growth failure, absent pubertal development, recurrent or atypical infections, and profound CD4+ T-cell depletion should prompt consideration of underlying immunodeficiency, including HIV. In patients with incomplete medical records, previous diagnoses should be reassessed, especially when the clinical course remains unexplained.

## 1. Introduction and Clinical Significance

Human immunodeficiency virus (HIV) infection in children and adolescents remains a significant global health challenge despite major advances in prevention and early diagnosis. With the widespread implementation of prevention of mother-to-child transmission programs, the incidence of pediatric HIV has markedly decreased; however, a considerable proportion of children and adolescents worldwide remain undiagnosed until late stages of the disease [1,2]. Recent data confirm that pediatric HIV continues to pose significant clinical and developmental challenges due to its complex immunological and multisystem impact [3]. Delayed diagnosis is particularly concerning because it is associated with increased morbidity, impaired growth and development, and a higher risk of opportunistic infections [4].

The clinical presentation of HIV infection acquired during childhood is highly heterogeneous and depends on the degree of immune suppression, viral replication, and coexisting conditions. While some infants develop severe disease early in life, others may exhibit a slower disease progression and may remain undiagnosed until adolescence [5]. In these cases, infection may present with nonspecific, multisystem involvement that may mimic other chronic conditions and lead to prolonged diagnostic delay [6]. Diagnostic delay may be particularly likely in patients with incomplete medical history or limited early-life documentation.

Growth failure and delayed puberty are among the most frequently reported manifestations in children and adolescents living with HIV. These abnormalities may result from a complex interplay of chronic inflammation, increased metabolic demands, nutritional deficiencies, chronic disease, and endocrine dysfunction [7,8,9]. As a consequence, clinicians may initially consider endocrine evaluation, while an underlying immunodeficiency may not be included in the differential diagnosis.

HIV infection may coexist with other infections and chronic conditions that further obscure the clinical picture. Such conditions may be regarded as the primary explanation for growth retardation, delayed pubertal development, or clinical abnormalities, thereby contributing to diagnostic delay. CMV exposure is common in immunocompromised patients, although CMV seropositivity alone does not establish active disease. In patients with advanced HIV infection, atypical or prolonged viral infections may also occur [10,11,12,13]. Growth retardation, delayed pubertal development, chronic organ abnormalities, and unusual or recurrent infections may therefore obscure the underlying diagnosis. Here, we report a case of an adolescent with previously undiagnosed advanced HIV infection, presenting with growth failure, delayed puberty, profound CD4+ T-cell depletion, and repeatedly positive SARS-CoV-2 tests, highlighting the diagnostic challenges associated with incomplete early-life medical history and diagnostic anchoring.

## 2. Case Presentation

A 16-year-old male was admitted to the Pediatrics Department of University Hospital Dr. Georgi Stranski-Pleven for evaluation of prolonged low-grade fever, chronic productive cough, and recurrent sinonasal symptoms.

The patient had a history of hepatosplenomegaly, persistently elevated liver enzymes, and marked delay in growth and pubertal development. The biological mother died during childbirth, and no antenatal or neonatal medical records were available. There was no documentation regarding maternal HIV testing, and her HIV status during pregnancy and at delivery could not be established. The boy was adopted at 4 years of age, with no accessible medical records from the pre-adoption period. No information about mode of infant feeding, neonatal HIV testing, or prophylaxis against mother-to-child transmission was available. Neurocognitive development was normal; however, growth retardation became evident, particularly after the age of 10 years.

Atopic dermatitis had been diagnosed in early childhood after adoption. No information on treatment with systemic corticosteroids or immunosuppressive agents was available. In October 2021, an appendectomy due to acute appendicitis was performed. At that time, hepatomegaly and elevated liver enzymes were first identified.

On current admission, the patient had marked growth retardation, with a height of 139 cm (−3.0 SDS) and a weight of 32 kg (−2.6 SDS), corresponding to measurements below the 3rd percentile for age and sex. General condition was mildly impaired.

At the time of admission, residual areas of hyper- and hypopigmentation were observed. Mild pharyngeal hyperemia, partial nasal obstruction, and productive cough were present. Chest auscultation demonstrated harsh vesicular breath sounds with crackles over the right subscapular region. No peripheral lymphadenopathy was detected. The liver was palpable 3–4 cm below the right costal margin, and the spleen 2–3 cm below the left costal margin. External genitalia were male, with prepubertal development (Tanner stage I). Despite the absence of pubertal progression by the age of 16, no formal endocrine evaluation had been performed. Therefore, an underlying endocrine disorder, including abnormalities of the growth hormone-insulin-like growth factor-1 or hypothalamic-pituitary-gonadal axes, could not be assessed. Neurological examination was normal.

### 2.1. Timeline

In October 2021, hepatomegaly and elevated liver enzymes were first documented during hospitalization for acute appendicitis. Initial investigations showed negative viral hepatitis serology and Epstein–Barr virus testing, normal C-reactive protein, and positive cytomegalovirus (CMV) IgG. Evaluation of peripheral eosinophilia excluded parasitic infections (echinococcosis, toxoplasmosis, toxocariasis), and allergy testing was negative.

In February 2022, the patient developed SARS-CoV-2 infection, documented by a positive SARS-CoV-2 rapid antigen test, with fatigue and persistent productive cough. In September 2022, a second SARS-CoV-2 rapid antigen test was documented, followed by intermittent subfebrile episodes. In October 2022, cranial computed tomography revealed chronic pansinusitis, and endoscopic sinus drainage was performed; microbiological cultures were negative. Fever recurred shortly after completion of postoperative antibiotic therapy with ceftriaxone, prompting further investigations for infectious, inflammatory, and metabolic causes.

During the subsequent diagnostic evaluation, additional SARS-CoV-2 antigen and PCR tests were reported as positive, while anti-SARS-CoV-2 antibodies remained undetectable. Because the complete chronological sequence of all SARS-CoV-2 tests and viral sequencing data were not available, the available findings do not allow definitive distinction between continuous viral persistence and separate infection or reinfection episodes of SARS-CoV-2.

A schematic timeline of the diagnostic process is shown in Table 1.

### 2.2. Diagnostic Assessment

Laboratory investigations demonstrated fluctuating leukocyte counts, mild anemia, intermittent thrombocytosis, and persistent elevation of hepatocellular enzymes. Aspartate aminotransferase (AST) was 304.6 U/L, alanine aminotransferase (ALT) 273.1 U/L, with elevated gamma-glutamyl transferase (GGT) and lactate dehydrogenase (LDH) up to 606 U/L. Inflammatory markers were within normal limits or minimally elevated (Table 2).

SARS-CoV-2 antigen and PCR tests were positive during the diagnostic course, while anti-SARS-CoV-2 antibodies were negative.

Immunophenotyping revealed severe CD4+ T-cell depletion, with CD4+ percentages ranging from 0.38% to 1.2% and absolute counts ranging from 4 to 42 cells/µL; the CD4/CD8 ratio ranged from 0.008 to 0.02 (Table 2).

HIV infection was confirmed by positive enzyme-linked immunosorbent assay (ELISA) and Western blot, with a plasma HIV RNA level of 127,721 copies/mL. Additional investigations, including viral hepatitis markers, Mantoux testing, leishmaniasis serology, throat cultures, and Candida testing, were negative (Table 3).

Chest radiography and thoracic computed tomography showed no focal inflammatory infiltrates.

Abdominal ultrasonography and computed tomography demonstrated hepatosplenomegaly without focal hepatic lesions or intraperitoneal fluid.

### 2.3. Diagnosis

Our findings were consistent with advanced HIV infection with severe immunosuppression. HIV infection was confirmed by positive ELISA and Western blot, together with a plasma HIV RNA level of 127,721 copies/mL. Because biological and medical records from infancy and early childhood were unavailable, the exact timing and route of HIV acquisition could not be established with certainty. However, the clinical course and advanced disease at adolescence were suggestive of longstanding infection, and acquisition during the perinatal period was considered possible, although not confirmed.

The profound depletion of CD4+ T lymphocytes and the clinical context were consistent with advanced HIV infection with severe immunosuppression. The repeated SARS-CoV-2 findings occurred in the setting of severe immunodeficiency, but the available data were insufficient to establish a single continuous SARS-CoV-2 infection.

## 3. Discussion

Pediatric HIV infection is characterized by a wide clinical spectrum, ranging from rapidly progressive disease in early childhood to slowly evolving infection that may remain unrecognized until adolescence. The present case illustrates a delayed diagnosis of advanced HIV infection amid multisystem involvement, in which several coexisting conditions and atypical clinical features obscured the underlying etiology. Contemporary evidence highlights pediatric HIV as a chronic multisystem disease with long-term developmental, immunological, and metabolic consequences, particularly in individuals diagnosed late [3]. In our patient, however, the route and timing of acquisition could not be confirmed because reliable early-life records were unavailable.

Advanced HIV infection frequently coexists with congenital or early-acquired infections. Among these, CMV infection is the most prevalent and clinically relevant. It may lead to chronic organ involvement, particularly affecting the liver, and may present with hepatomegaly and persistent elevation of transaminases [14,15]. Recent studies emphasize that early-life HIV–CMV coinfection is not only frequent but also associated with synergistic immunopathological effects, including enhanced immune activation, accelerated immunosenescence, and worse clinical outcomes [16]. Furthermore, CMV infection in HIV-infected children has been associated with more severe clinical presentations, including pulmonary and systemic involvement, reinforcing its role as both a comorbidity and a modifier of disease progression [17]. In the current case, CMV exposure was considered during the earlier diagnostic evaluation because CMV-specific IgG antibodies were detected in the setting of hepatomegaly and elevated liver enzymes. However, CMV IgG positivity alone indicates previous exposure and does not establish active CMV infection or CMV-related hepatic disease. No contemporaneous CMV DNA/PCR, antigenemia, histopathological evidence, or other definitive evidence of active CMV disease was available. Therefore, CMV infection should be regarded as a previous clinical attribution rather than a confirmed cause of the hepatic abnormalities in this patient. Nevertheless, the earlier attribution to CMV may have contributed to diagnostic anchoring and delayed consideration of an underlying immunodeficiency.

HIV infection is associated with progressive impairment of both innate and adaptive immunity, resulting in increased susceptibility to a broad range of opportunistic and non-opportunistic infections. Patients may experience more frequent, severe, recurrent, or atypical infections compared to immunocompetent individuals [18]. In the context of severe CD4+ T-cell depletion, infections may also exhibit prolonged or incomplete resolution because effective cellular immune responses are impaired. The repeated detection of SARS-CoV-2 in our patient occurred in the setting of profound cellular immunodeficiency. Although prolonged SARS-CoV-2 infection has been described in severely immunocompromised individuals [11,12,13], the available clinical and laboratory data in this case do not allow us to establish with certainty whether the repeated positive results represented continuous viral persistence or separate infection/reinfection episodes. In this context, the absence of detectable SARS-CoV-2-specific antibodies despite documented positive tests may reflect impaired antigen-specific responses and further underscores the immune system’s inability to mount an effective, antigen-specific humoral response in advanced HIV infection. Therefore, the SARS-CoV-2 findings should be interpreted as evidence of repeated or prolonged viral detection rather than definitive proof of a single continuous infection.

Another important aspect of HIV infection is the increased risk of malignancies, particularly those associated with oncogenic viruses, such as Epstein–Barr virus-related lymphomas and Kaposi sarcoma [19]. Although no neoplastic disease was identified in the present case, the underlying mechanisms—chronic immune activation, impaired immune surveillance, and viral co-infections are already present and highlight the long-term risks associated with delayed diagnosis.

In addition to immune-mediated manifestations, HIV infection has significant systemic effects that are not directly attributable to opportunistic infections.

Growth failure and delayed pubertal development are recognized clinical features in children and adolescents living with chronic HIV infection and may result from multiple interacting factors, including chronic inflammation, nutritional impairment, chronic disease, and endocrine dysfunction [7,8]. Recent data also indicate that growth impairment may persist into late adolescence among individuals with HIV acquired during the perinatal period [9]. In the present case, severe growth retardation and absent pubertal development were prominent clinical findings. However, because a formal endocrine evaluation was not performed, a specific endocrine cause could not be excluded, and a direct causal relationship between HIV infection and the growth and pubertal abnormalities cannot be established. These findings should therefore be regarded as important clinical manifestations occurring in the context of advanced HIV infection rather than as proven endocrine consequences of HIV. The immunological findings in this patient are highly characteristic of advanced HIV infection and played a pivotal role in establishing the diagnosis. The profound depletion of CD4+ T lymphocytes, along with a markedly reduced CD4/CD8 ratio, reflects severe cellular immune dysfunction and is a well-established marker of disease progression [20]. In parallel, the patient exhibited increased concentrations of several immunoglobulin classes, including IgG, IgA, IgM, and IgE. This pattern is compatible with polyclonal B-cell activation described in chronic HIV infection [21]. Importantly, increased quantitative immunoglobulin concentrations do not necessarily indicate effective antigen-specific immunity. The absence of detectable antibodies against SARS-CoV-2 in our patient, despite documented viral detection, illustrates the potential dissociation between quantitative immunoglobulin levels and specific humoral responses.

A central theme emerging from this case is the delay in diagnosis and the factors contributing to it. Retrospectively, multiple clinical indicators—including persistent growth failure, delayed puberty, chronic liver abnormalities, and atypical infectious patterns—could have prompted earlier investigation for underlying immunodeficiency. However, several factors contributed to the delayed recognition of HIV infection. First, the presence of a plausible alternative diagnosis, namely presumed CMV-related disease, together with repeatedly positive SARS-CoV-2 tests, may have led to diagnostic anchoring and reduced consideration of other etiologies. Published reports of prolonged SARS-CoV-2 infection in patients with advanced HIV remain limited and consist predominantly of individual case reports and small series [22,23]. Second, the absence of severe or life-threatening infections in early childhood may have lowered clinical suspicion for immunodeficiency. Third, the incomplete medical history due to adoption at the age of four years limited access to crucial perinatal information, including maternal HIV status and early screening results. Together, these factors illustrate how both clinical reasoning biases and gaps in medical history can significantly impact diagnostic pathways. This case emphasizes the importance of maintaining a high index of suspicion for HIV infection in children and adolescents presenting with multisystem involvement, even when individual findings appear to have alternative explanations. Particular attention should be given to the combination of severe growth and pubertal delay, chronic or unexplained organ abnormalities, recurrent or atypical infections, and characteristic immunological abnormalities. The absence of a reliable early-life history should not be interpreted as evidence against longstanding infection.

## 4. Limitations

This case report has several limitations. First, reliable antenatal, neonatal, and early-childhood medical records were unavailable, preventing confirmation of the timing and route of HIV acquisition. Second, complete longitudinal SARS-CoV-2 PCR results, cycle-threshold values, viral cultures, and genomic sequencing were unavailable; therefore, continuous viral persistence could not be distinguished definitively from repeated infection or reinfection. Third, CMV IgG positivity did not establish active CMV disease, and contemporaneous CMV PCR or other definitive evidence of CMV-related hepatic disease was unavailable. Fourth, formal endocrine evaluation, including assessment of the growth hormone/IGF-1 and hypothalamic-pituitary-gonadal axes and bone age, was not performed, limiting interpretation of the growth failure and delayed puberty. Finally, detailed antiretroviral treatment and longitudinal virological, immunological, growth, and pubertal outcomes were unavailable.

## 5. Future Directions

Future studies should investigate the interaction between advanced HIV-associated immune dysfunction, growth and pubertal development, and prolonged or recurrent SARS-CoV-2 infection using longitudinal clinical, endocrine, immunological, and virological assessments. In patients with prolonged SARS-CoV-2 positivity, serial PCR testing, cycle-threshold measurements, viral sequencing, and assessment of virus-specific immune responses may help distinguish persistent infection from reinfection and clarify the mechanisms underlying prolonged viral detection.

## 6. Conclusions

This case highlights the diagnostic complexity of advanced HIV infection presenting during adolescence with severe growth failure, absent pubertal development, profound CD4+ T-cell depletion, and multisystem clinical abnormalities. The absence of reliable early-life medical records contributed to uncertainty regarding the timing and route of HIV acquisition, although the clinical presentation was suggestive of longstanding infection.

Repeatedly positive SARS-CoV-2 tests occurred in the setting of severe immunodeficiency; however, the available data do not allow definitive distinction between persistent infection and repeated infection or reinfection. Similarly, previous CMV IgG positivity did not establish active CMV disease or CMV-related hepatic involvement.

This report underscores that adolescents presenting with severe growth or pubertal abnormalities, unexplained organ involvement, recurrent or atypical infections, and marked immune abnormalities should be evaluated for underlying immunodeficiency, including HIV. In patients with incomplete medical histories, previous diagnoses should be periodically reassessed when the clinical course is not fully explained by the presumed diagnosis.

## Figures and Tables

**Table 1:** Schematic timeline of case development and diagnosis.

Time	Clinical Findings	Investigations/Diagnosis	Intervention/Outcome
Early childhood	Atopic dermatitis; progressive growth retardation	Early-life records unavailable	Timing of HIV acquisition could not be established
Oct 2021	Acute appendicitis; hepatomegaly	Elevated liver enzymes; CMV IgG positive; viral hepatitis/EBV negative	Appendectomy; hepatic abnormalities persisted
Feb–Sep 2022	Fatigue, cough, subfebrile episodes	Repeated SARS-CoV-2 positivity	Further evaluation
Oct 2022	Chronic sinonasal symptoms and fever	CT: chronic pansinusitis; cultures negative	Endoscopic drainage + ceftriaxone; fever recurred
Subsequent evaluation	Severe growth failure, Tanner I, hepatosplenomegaly	HIV ELISA/Western blot positive; HIV RNA 127,721 copies/mL; CD4 4–42 cells/µL	Advanced HIV infection diagnosed

**Table 2:** Hematological, immunological, and biochemical parameters.

Parameter	Reference Ranges	Unit	07.11	08.11	16.11	21.11	23.11
WBC	4.0–10.0	×10^9^/L	5.7	3.88	2.6	5.6	5.2
RBC	4.30–5.70	×10^12^/L	4.32	3.92	3.67	4.19	3.77
HGB	130–160	g/L	119.0	104.0	105.0	121.0	103.0
HCT	0.41–0.52	L/L	0.36	0.322	0.301	0.359	0.297
MCV	78–98	fL	83.3	82.2	82.1	85.5	78.7
MCH	27–33	pg	27.7	26.6	28.8	28.8	27.3
Lym	20–45	%	42.5	28.3	31.3	36.0	19.1
Gran	40–75	%	47.0	55.8	59.4	54.8	73.4
Mo	2–10	%	10.5	8.8	9.3	9.2	7.5
PLT	139–320	×10^9^/L	398.0	410.0	353.0	267.0	386.0
CRP	<5.0	mg/L	0.16			0.06	
AST	8–48	U/L	170.1		304.6		58.7
ALT	7–55	U/L	164.0		273.1		49.7
GGT	<43	U/L	98.0		81.0		
ALP	82–331	U/L	121.0		109.0		
LDH	105–233	U/L					606
Fibrinogen	2.0–4.0	g/L	2.41			2.41	2.37
Total T lymphocytes	20–48	%				55.45	58.82
CD4+ T-helper cells	32.6–51.5	%				1.20	0.38
CD8+ cytotoxic suppressor T cells	27.0–35.0	%				54.45	45.47
CD4/CD8 ratio	1.1–4.4	ratio				0.02	0.008

**Table 3:** Microbiological, virological, and serological investigations.

Parameter	Reference Ranges	Unit	Result
SARS-CoV-2 rapid antigen test			positive
SARS-CoV-2 PCR			positive
IgG/IgM antibodies against SARS-CoV-2			negative
IgE	≤100	IU/mL	1760.8
IgM	0.4–2.5	g/L	2.93
IgG	6.0–16.0	g/L	21.45
IgA	0.8–4.0	g/L	5.39
C3	0.80–1.8	g/L	1.00
C4	0.15–0.45	g/L	0.22
MPO (pANCA)	<3.5	U/mL	1.5
PR3/cANCA	<3.5	U/mL	2.3
ANA			negative
Throat culture			negative
Serology for visceral leishmaniasis			negative
Mantoux test			negative
Hepatitis markers			negative
Oral Candida			negative
Fecal Candida			negative
HIV antibodies–ELISA			positive
HIV antibodies–Western blot			positive
HIV RNA		copies/mL	127,721
Influenza type A/B			negative

## Data Availability

All original data presented in this study are included in the article. Further inquiries can be directed to the corresponding author.

## Abbreviations

The following abbreviations are used in this manuscript:

HIV	Human Immunodeficiency Virus
CMV	Cytomegalovirus

## References

[1] World Health Organization Global HIV & AIDS Statistics—Fact Sheet. https://www.who.int/news-room/fact-sheets/detail/hiv-aids.

[2] UNICEF Children, HIV and AIDS: Global Snapshot 2022. https://www.unicefusa.org/press/despite-progress-adolescent-girls-continue-bear-brunt-hiv-epidemic-98000-new-infections-2022.

[3] Nowak K., Łupina K., Lorek D., Jabłońska A., Radomska A., Choromański T., Janczura J. (2025). Pediatric Human Immunodeficiency Virus Infection in the Modern Era: A Systematic Review of Recent Advances and Their Implications. Pediatr. Investig..

[4] Vreeman R.C., Rakhmanina N.Y., Nyandiko W.M., Puthanakit T., Kantor R. (2021). Are We There Yet? 40 Years of Successes and Challenges for Children and Adolescents Living with HIV. J. Int. AIDS Soc..

[5] Marston M., Becquet R., Zaba B., Moulton L.H., Gray G., Coovadia H., Essex M., Ekouevi D.K., Jackson D., Coutsoudis A. (2011). Net Survival of Perinatally and Postnatally HIV-Infected Children: A Pooled Analysis of Individual Data from Sub-Saharan Africa. Int. J. Epidemiol..

[6] Ferrand R.A., Corbett E.L., Wood R., Hargrove J., Ndhlovu C.E., Cowan F.M., Gouws E., Williams B.G. (2009). AIDS Among Older Children and Adolescents in Southern Africa: Projecting the Time Course and Magnitude of the Epidemic. AIDS.

[7] Arpadi S.M. (2000). Growth Failure in Children with HIV Infection. J. Acquir. Immune Defic. Syndr..

[8] Rogol A.D., Roemmich J.N., Clark P.A. (2002). Growth at Puberty. J. Adolesc. Health.

[9] Costa P.R.F., Guimarães N.S., Lira C.R.N., Leite L.O., de Cássia Ribeiro da Silva R., Barreto M.L., Paixão E.S. (2025). Growth and Body Composition of Adolescents and Young Adults with Perinatal HIV Infection: A Systematic Review and Meta-Analysis. BMC Public Health.

[10] Pinninti S., Arora N., Boppana S. (2026). Cytomegalovirus and HIV Co-Infections during Pregnancy and Early Childhood. Curr. HIV/AIDS Rep..

[11] Peluso M.J., Antar A.A.R. (2023). Long COVID in People Living with HIV. Curr. Opin. HIV AIDS.

[12] Choi B., Choudhary M.C., Regan J., Sparks J.A., Padera R.F., Qiu X., Solomon I.H., Kuo H.H., Boucau J., Bowman K. (2020). Persistence and Evolution of SARS-CoV-2 in an Immunocompromised Host. N. Engl. J. Med..

[13] Avanzato V.A., Matson M.J., Seifert S.N., Pryce R., Williamson B.N., Anzick S.L., Barbian K., Judson S.D., Fischer E.R., Martens C. (2020). Case Study: Prolonged Infectious SARS-CoV-2 Shedding from an Asymptomatic Immunocompromised Individual with Cancer. Cell.

[14] Dollard S.C., Grosse S.D., Ross D.S. (2007). New Estimates of the Prevalence of Neurological and Sensory Sequelae and Mortality Associated with Congenital Cytomegalovirus Infection. Rev. Med. Virol..

[15] Manicklal S., Emery V.C., Lazzarotto T., Boppana S.B., Gupta R.K. (2013). The “Silent” Global Burden of Congenital Cytomegalovirus. Clin. Microbiol. Rev..

[16] Albano C., Gugliesi F., Bajetto G., Braga B., Dell’Oste V., Griffante G., Pasquero S. (2026). The Long Shadow of Early HCMV-HIV Coinfection: Epidemiology, Pathogenesis, and Immune Consequences. Children.

[17] Tsanglao W.R., Joshi M., Aier T., Kikon S. (2024). Severe Pneumonia and Cytomegalovirus Coinfection in Infants with Human Immunodeficiency Virus Infection. Indian J. Public Health.

[18] Kaplan J.E., Benson C., Holmes K.K., Brooks J.T., Pau A., Masur H., Centers for Disease Control and Prevention (CDC), National Institutes of Health, HIV Medicine Association of the Infectious Diseases Society of America (2009). Guidelines for Prevention and Treatment of Opportunistic Infections in HIV-Infected Adults and Adolescents: Recommendations from CDC, the National Institutes of Health, and the HIV Medicine Association of the Infectious Diseases Society of America. MMWR Recomm. Rep..

[19] Shiels M.S., Pfeiffer R.M., Gail M.H., Hall H.I., Li J., Chaturvedi A.K., Bhatia K., Uldrick T.S., Yarchoan R., Goedert J.J. (2011). Cancer Burden in the HIV-Infected Population in the United States. J. Natl. Cancer Inst..

[20] Lu W., Mehraj V., Vyboh K., Cao W., Li T., Routy J.P. (2015). CD4:CD8 Ratio as a Frontier Marker for Clinical Outcome, Immune Dysfunction and Viral Reservoir Size in Virologically Suppressed HIV-Positive Patients. J. Int. AIDS Soc..

[21] Moir S., Fauci A.S. (2009). B Cells in HIV Infection and Disease. Nat. Rev. Immunol..

[22] Velasquez-Reyes J.M., Schaeffer B., Curry S.R., Overbeck V., Sher-Jan C., Taylor B.P., Turcinovic J., Kuppalli K., Connor J.H., Hanage W.P. (2025). Characterisation of a Persistent SARS-CoV-2 Infection Lasting More than 750 Days in a Person Living with HIV: A Genomic Analysis. Lancet Microbe.

[23] Kawashima A., Tsuchiya K., Kuroki K., Nagashima M., Koizumi Y., Nakamoto T., Mizushima D., Aoki T., Sadamasu K., Teruya K. (2026). Prolonged Severe Acute Respiratory Syndrome Coronavirus 2 Infection and Intrahost Viral Evolution in a Severely Immunocompromised Patient with Human Immunodeficiency Virus Infection: A Whole-Genome Sequencing Study. Int. J. Infect. Dis..

